# Low-Melting Manganese(II)-Based Ionic Liquids: Syntheses, Structures, Properties and Influence of Trace Impurities

**DOI:** 10.3390/ma12223764

**Published:** 2019-11-15

**Authors:** Tim Peppel, Monika Geppert-Rybczyńska, Christin Neise, Udo Kragl, Martin Köckerling

**Affiliations:** 1 Leibniz-Institut für Katalyse e.V. (LIKAT), Heterogene Photokatalyse, Albert-Einstein-Str. 29a, 18059 Rostock, Germany; 2 Instytut Chemii, Uniwersytet Śląski w Katowicach, Szkolna 9, 40-006 Katowice, Poland; monika.geppert-rybczynska@us.edu.pl; 3 Institut für Chemie, Anorganische Festkörperchemie, Universität Rostock, Albert-Einstein-Str. 3a, 18059 Rostock, Germany; christin.neise@gmx.de; 4 Institut für Chemie, Technische Chemie, Universität Rostock, Albert-Einstein-Str. 3a, 18059 Rostock, Germany; udo.kragl@uni-rostock.de; 5 Department Life, Light and Matter, Universität Rostock, 18051 Rostock, Germany; 6 Leibniz-Institut für Katalyse e.V. (LIKAT), Polymerchemie und Katalyse, Albert-Einstein-Str. 29a, 18059 Rostock, Germany

**Keywords:** manganese, ionic liquid, crystal structure, physical properties, trace impurities

## Abstract

The synthesis of more than 10 new magnetic ionic liquids with [Mn*X*_4_]^2−^ anions, *X* = Cl, NCS, NCO, is presented. Detailed structural information through single-crystal X-ray diffraction is given for (DMDIm)[Mn(NCS)_4_], (BnEt_3_N)_2_[Mn(NCS)_4_], and {(Ph_3_P)_2_N}_2_[Mn(NCO_4_)]·0.6H_2_O, respectively. All compounds consist of discrete anions and cations with tetrahedrally coordinated Mn(II) atoms. They show paramagnetic behavior as expected for spin-only systems. Melting points are found for several systems below 100 °C classifying them as ionic liquids. Thermal properties are investigated using differential scanning calorimetry (DSC) measurements. The physicochemical properties of density, dynamic viscosity, electrolytic conductivity, and surface tension were measured temperature-dependent of selected samples. These properties are discussed in comparison to similar Co containing systems. An increasing amount of bromide impurity is found to affect the surface tension only up to 3.3%.

## 1. Introduction

For the last 20 years, the ongoing tremendous interest in ionic liquids (ILs) as salts with low melting points is based on their partly unique properties which lead to a variety of possible applications. Therefore, ILs have been thoroughly investigated as systems with large electrochemical windows and liquid ranges, hardly measurable vapor pressures, unusual solubility characteristics, and applications in catalysis [1,2,3,4,5,6,7,8,9,10,11,12,13,14,15]. ILs containing metal-based complex ions, so-called magnetic ionic liquids (MILs), are a highly investigated subclass of ILs, because they show magnetic response in addition to the characteristics mentioned above [16,17,18,19,20]. Such ILs with paramagnetic transition-metal complex anions have also been assumed to be magnetic and magnetorheological fluids [21,22,23].

In the last 10 years, we have reported on different classes of transition metal-containing low-melting ILs with complex anions of the 3d metals Cr, Co, and Ni, respectively, of which a series of physicochemical data is available [24,25,26,27,28,29,30,31,32,33,34]. The astonishing results from imidazolium-based ILs containing the [Co(NCS)_4_]^2−^ anion [25] led us to extending our research for new low-melting, transparent ILs which contain tetrahedrally coordinated manganese(II) complex anions which could also be suitable as catalysts in chemical reactions. Since the structural motifs especially for manganese complexes is inexhaustible due to the wide range of possible oxidation states (+I to +VII), our research has been focused on homoleptic Mn(II) complex anions only for better comparison with already known MIL subclasses.

The subclass of compounds with (pseudo)tetrahedrally coordinated [Mn^II^*X*_4_]^2−^ (*X* = halide, SCN, OCN) complex anions has been known for more than 100 years [35,36,37,38,39,40,41,42,43], but crystal and molecular structure investigations are still scarce. In order to get a better understanding of solid-state properties of these salts, it is necessary to investigate their crystal and molecular structures. There has been a study on manganate(II)-based ionic liquids with main focus on physico-optical properties of [Mn^II^*X*_4_]^2−^-based systems (*X* = Cl, Br, NTf_2_ [NTf_2_: bis (trifluoromethanesulfonyl) amido]) which are solids at room temperature and no single crystal data has been presented [44].

To the best of our knowledge, crystallographic investigations of imidazolium-based salts containing the [Mn^II^*X*_4_]^2−^ (*X* = halide) anions are only known for (ImH)_2_[MnCl_4_]·(ImH)_4_[MnCl_6_] (ImH = 1 H-imidazolium) [45], (MImPPy)[MnCl_4_] (MImPPy = 1-(3-(1-Methylimidazolium-3-yl) propyl) pyridinium) and (DMBIm)[MnCl_4_] (DMBIm = 1,1’-Butane-1, 4-diylbis (3-methylimidazolium) [46], (BMIm)_2_[MnCl_4_] (BMIm = 1-Butyl-3-methylimidazolium) [47,48] and (BMIm)_2_[MnBr_4_] [49], respectively. It is obvious that, until now, there has been no single crystal data available for imidazolium-based systems, which contain pseudohalides as ligands in the complex anion [Mn^II^(NCX)_4_]^2−^ (*X* = O, S). There are only few reports on crystal structures of [Mn^II^(NCS)_4_]^2−^ containing systems [50,51,52,53], and only one single report on the crystal structure of a [Mn^II^(NCO)_4_]^2−^ containing system, [Ni(bpy)_3_][Mn(NCO)_4_]·H_2_O (bpy = 2,2′-bipyridine) [54], respectively.

In this contribution, we report on the synthesis, properties and physicochemical investigation of a series of A_x_[Mn*X*_4_] (A = ammonium-, iminium- and imidazolium-based cation; *X* = Cl, NCS, NCO) complexes with comparably low melting points. The series was divided into two subclasses: a) non-hygroscopic A_x_[Mn(NCS)_4_] compounds, and b) slightly hygroscopic A_x_[Mn*X*_4_] (*X* = Cl, NCO) compounds, respectively. All compounds were investigated by means of elemental analysis and mid-infrared (MIR) spectroscopy. The first subclass was further investigated by nuclear magnetic resonance (NMR) spectroscopy to obtain magnetic data. Additional measurements of density, dynamic viscosity, conductivity, and surface tension were done exemplarily for the low-melting non-hygroscopic substance (EMIm)_2_[Mn(NCS)_4_] (EMIm = 1-ethyl-3-methylimidazolium). The influence of trace impurities (residual bromide) is also discussed on the basis of the physicochemical properties. Electronic spectra are not presented in detail due to the already existing large amount of such data [33,34,36,55]. The crystal and molecular structures of three compounds, (DMDIm)[Mn(NCS)_4_], (BnEt_3_N)_2_[Mn(NCS)_4_], and {(Ph_3_P)_2_N}_2_[Mn(NCO_4_)]·0.6H_2_O, respectively, are also presented and discussed.

## 2. Experimental

### 2.1. Instrumentation

NMR spectra were recorded on a Avance 250 or Avance 300 spectrometer (Bruker, Billerica, MA, USA) and chemical shifts of the ^1^H, and ^13^C spectra were reported in parts per million (ppm) using the solvent shifts for ^1^H and ^13^C as internal standard ([D6]DMSO: ^1^H δ = 2.50 ppm, ^13^C δ = 39.5 ppm; D_2_O: ^1^H δ = 4.75 ppm; CDCl_3_: ^1^H *δ* = 7.26, ^13^C *δ* = 77.0) [55]. Elemental analysis for C, H, N and S was performed on a FlashEA 1112 device (Thermo Fisher Scientific, Waltham, MA, USA). The MIR spectra were recorded by the attenuated total reflectance (ATR) technique on a Nicolet 380 FT-IR spectrometer (Thermo Fisher Scientific, Waltham, MA, USA). Melting points were determined by differential scanning calorimetry (DSC) measurements with a DSC823^e^ instrument (Mettler-Toledo, Columbus, OH, USA) in the range −100 to 200 °C at a heating rate of 10 K min^−1^ (Ar atmosphere, Al crucible) or with a STA 449 F3 Jupiter device (Netzsch, Selb, Germany) in the temperature range 25 to 600 °C at a heating rate of 10 K min^−1^ (N_2_ atmosphere, Al crucible), respectively. Single-crystal X-ray diffraction measurements were made with a Apex X8 diffractometer (Bruker-Nonius, Billerica, MA, USA) equipped with a CCD detector. The measurements were performed with monochromatic Mo-Kα radiation (λ = 0.71073 Å). The preliminary unit-cell data were obtained from the reflection positions of 36 frames, measured in different directions of reciprocal space. After the completion of the data measurements, the intensities were corrected for Lorentz, polarization, and absorption effects with the Bruker-Nonius software [56]. The structure solutions and refinements were performed with the SHELX-97 program package [57]. All of the non-hydrogen atoms were refined anisotropically. The hydrogen atoms were added at idealized positions and refined in riding models. These data can be obtained free of charge from the Cambridge Crystallographic Data Centre: CCDC 834685 for (DMDIm)[Mn(NCS)_4_], CCDC 834684 for (BnEt_3_N)_2_[Mn(NCS)_4_], and CCDC 1934913 for {(Ph_3_P)_2_N}_2_[Mn(NCO_4_)]·0.6H_2_O. Magnetic data were determined by ^1^H NMR spectroscopy techniques [58,59]. Molar susceptibilities were corrected by applying Pascal constants [60]. Effective magnetic moments μ_eff_/μ_B_ were determined by applying the Langevin equation [61]. Trace impurities ([pseudo-]halides, sulphate) were determined by an ionic chromatography (IC) system equipped with a Alltech 550 model conductivity detector. A Metrosep Anion Dual 2 75/4.6 mm column was employed and the mobile phase consisted of a mixture of 1.3 mmol/L NaHCO_3_ and 2.0 mmol/L Na_2_CO_3_ in pure water with a flow rate of 0.8 mL/min. The column temperature was 25 °C. For sample preparation approximately 100 mg of ionic liquid was weighed into a 100 mL volumetric flask and dissolved in pure water (1:1000). The solution was transferred to a sample vial, manually injected and analysed with the IC system. Water contents were determined by Karl Fischer titration on a TitroLine KF Trace device (SI Analytics, Weilheim, Germany). Densities (*ρ*) and dynamic viscosities (*η*) were measured with an Stabinger viscometer SVM 3000 (Anton Paar, Graz, Austria). The specification provided by the manufacturer estimates the reproducibility as 0.35% for viscosities and 5 × 10^−4^ g∙cm^−3^ for densities with repeatability on the level 0.1% and 2 × 10^−4^ g∙cm^−3^ for viscosity and density, respectively. Surface tensions (*γ*) were measured using the pendant drop method with the DSA 10 Tensiometer with Drop Shape Analysis software (Krüss GmbH, Hamburg, Germany). The instrumental procedures and the experimental details have been published previously [34,62]. The general uncertainty of the method given by the manufacturer is 0.1 mN∙m^−1^. Conductivities (*κ*) were measured with a Microprocessor Conductivity Meter LF 537 with a Tetracon 96 electrode with the general uncertainty 0.5% (WTW, Weilheim, Germany).

### 2.2. Materials

All commercially available chemicals were used as received (Sigma-Aldrich, St. Louis, MO, USA, purities >99%). *N*-methylimidazole was freshly distilled from KOH in vacuo before use. Monocationic ionic liquid precursors were prepared by literature procedures [63,64].

K_2_[Mn(NCS)_4_] was synthesized on a multi-gram level: Manganese(II) chloride (MnCl_2_, 10.0 g, 79.5 mmol), potassium thiocyanate (KSCN, 31.7 g, 325.8 mmol) were placed in a round-bottom flask and heated under reflux overnight in 250 mL acetone. The precipitate (KCl) was filtered off and the solvent of the light green filtrate was completely removed in vacuo. The residue was extracted with 100 mL dichloromethane, filtered and the solvent was removed again in vacuo. The purified solid was dried overnight at 120 °C, yielding an almost white hygroscopic powder in high yield (26.5 g, 72.5 mmol, 91%). M.p. 169 °C; elemental analysis for C_4_K_2_MnN_4_S_4_: calcd. C 13.2, N 15.3, S 35.1; found C 15.2, H 0.5, N 14.4, S 36.7.

AgOCN was freshly precipitated each time prior to use by direct salt metathesis of KOCN with excess AgNO_3_ in aqueous solution at room temperature in quantitative yield. The resulting colorless precipitate was filtered off, thoroughly washed with water, ethanol, diethyl ether and finally dried at 60 °C. Elemental analysis for CAgNO: calcd. C 8.0, N 9.4; found C 7.7, H 0.1, N 9.6.

### 2.3. Synthesis of A_n_^x+^[Mn(NCS)_4_]^2−^

Samples of the general formula A_n_^x+^[Mn(NCS)_4_]^2−^ (n = 2, x = 1: A = (BnEt_3_N)^+^, {(Ph_3_P)_2_N}^+^, (EMIm)^+^, (BMIm)^+^; n = 1, x = 2: A = (DMDIm)^2+^) were prepared by adaptation of a known literature procedure [65] via salt metathesis reaction of 2.05 eq. *A*^+^*X*^−^ (2.05 mmol) (*A*^+^*X*^−^_=_ (BnEt_3_N)Cl, {(Ph_3_P)_2_N}Cl, (EMIm)Br, (BMIm)Br) or 1.05 eq. *A*^2+^*X*_2_^−^ (1.05 mmol) (*A*^2+^*X*_2_^−^ = (DMDIm)Cl_2_) and 1.00 eq. K_2_[Mn(NCS)_4_] (1.00 mmol) in 50 mL boiling acetone. The resulting suspensions were refluxed overnight, filtered, afterwards the solvent was removed in vacuo and the residue was washed with portions of dichloromethane. The solvent of the combined filtrates was removed in vacuo yielding light greenish compounds in good yields (>75%).

**(BnEt_3_N)_2_[Mn(NCS)_4_]**, bis (benzyl-triethylammonium) tetraisothiocyanatomanganate(II): Prepared from commercial (BnEt_3_N)Cl. Light green solid, yield 90%. M.p. (exp.) 101 °C, glass transition (exp.) −13 °C, T_g_/T_m_ = 0.70; elemental analysis for C_30_H_44_MnN_6_S_4_ (calcd.): C 54.4 (53.6), H 7.1 (6.6), N 12.4 (12.5), S 18.8 (19.0); IR (ATR, 32 scans, 25 °C, cm^−1^): 3032, 3005, 2985, 2945, 2873, 2069 (CN str. [65]), 2030, 1495, 1477, 1454, 1392, 1365, 1328, 1211, 1152, 1082, 1007, 966, 941, 902, 829 (CS str. [65]), 811, 793, 776, 751, 703, 604, 536.

**{(Ph_3_P)_2_N}_2_[Mn(NCS)_4_]**, bis-(bis (triphenylphosphine) iminium) tetraisothiocyanatomanganate(II): Prepared from commercial {(Ph_3_P)_2_N}Cl. Greenish solid, yield 94%. M.p. (exp.) 144 °C, glass transition (exp.) 55 °C, T_g_/T_m_ = 0.79; elemental analysis for C_76_H_60_MnN_6_P_4_S_4_ (calcd.): C 66.7 (66.9), H 4.4 (4.4), N 6.2 (6.2), S 9.5 (9.4); IR (ATR, 32 scans, 25 °C, cm^−1^): 3144, 2983, 2955, 2872, 2037 (CN str. [65]), 1567, 1444, 1430, 1330, 1290, 1261, 1163, 1100, 1090, 960, 825 (CS str. [65]), 739, 700, 643, 620, 598.

**(EMIm)_2_[Mn(NCS)_4_]**, bis (1-ethyl-3-methylimidazolium) tetraisothiocyanatomanganate(II): Prepared from (EMIm)Br [66]. Green liquid, yield 76%. glass transition (exp.) −57 °C, M.p. (calcd.) 51 °C; elemental analysis for C_16_H_22_MnN_8_S_4_ (calcd.): C 37.5 (37.7), H 4.1 (4.3), N 20.6 (21.9), S 22.3 (25.1); IR (ATR, 32 scans, 25 °C, cm^−1^): 3145, 3103, 2982, 2953, 2874, 2036 (CN str. [65]), 1565, 1446, 1425, 1385, 1334, 1291, 1261, 1163, 1105, 1086, 1029, 958, 827 (CS str. [65]), 740, 700, 644, 618, 596; *μ*_eff_ = 5.9 *μ*_B_ (*T* = 25 °C, *c* = 49.0 mmol/L, *υ*_0_ = 300 MHz, *χ*_mol_ = 14.6 × 10^−3^).

**(BMIm)_2_[Mn(NCS)_4_]**, bis (1-butyl-3-methylimidazolium) tetraisothiocyanatomanganate(II): Prepared from (BMIm)Br [67]. Green liquid, yield 77%. glass transition (exp.) −58 °C, M.p. (calcd.) 50 °C; elemental analysis for C_24_H_30_MnN_8_S_4_ (calcd.): C 43.6 (42.5), H 5.6 (5.4), N 19.7 (19.8), S 22.9 (22.7); IR (ATR, 32 scans, 25 °C, cm^−1^): 3143, 3104, 2958, 2932, 2872, 2040 (CN str. [65]), 1563, 1461, 1425, 1380, 1336, 1278, 1207, 1162, 1105, 1087, 1022, 963, 824 (CS str. [65]), 740, 696, 647, 618; *μ*_eff_ = 6.0 *μ*_B_ (*T* = 25 °C, *c* = 42.4 mmol/L, *υ*_0_ = 300 MHz, *χ*_mol_ = 15.1 × 10^−3^).

**(DMDIm)[Mn(NCS)_4_]**, 3,3′-methylenbis (1-methyl-imidazolium) tetraisothiocyanatomanganate(II): Prepared from (DMDIm)Cl_2_ [34]. Light green solid, yield 86%. M.p. (exp.) 179 °C, glass transition (exp.) 26 °C, T_g_/T_m_ = 0.66; elemental analysis for C_13_H_14_MnN_8_S_4_ (calcd.): C 30.7 (33.5), H 2.5 (3.0), N 22.0 (24.0), S 27.5 (27.6); IR (ATR, 32 scans, 25 °C, cm^−1^): 3150, 3130, 3098, 3070, 2092, 2054 (CN str. [65]), 1699, 1599, 1558, 1546, 1427, 1367, 1321, 1234, 1160, 1081, 958, 838, 827 (CS str. [65]), 765, 749, 732, 673, 620, 602, 536.

### 2.4. Synthesis of A_n_^x+^[MnCl_4_]^2−^

Samples of the general formula A_2_^+^[MnCl_4_]^2−^ (A = (BnEt_3_N)^+^, {(Ph_3_P)_2_N}^+^, (EMIm)^+^, (BMIm)^+^) were prepared by adaptation of a known literature procedures [37,41] via direct reaction of 2.05 eq. *A*^+^*X*^−^ (2.05 mmol) (*A*^+^*X*^−^ = (BnEt_3_N)Cl, {(Ph_3_P)_2_N}Cl, (EMIm)Cl, (BMIm)Cl) or 1.05 eq. *A*^2+^*X*_2_^−^ (1.05 mmol) (*A*^2+^*X*_2_^−^ = (DMDIm)Cl_2_) and 1.00 eq. anhydrous MnCl_2_ (1.00 mmol) in 50 mL boiling ethanol. The resulting suspensions were refluxed for 30 min, cooled down to room temperature and the solvent was removed in vacuo. The residue was washed thoroughly with portions of ethyl acetate/ethanol (1:1) and diethyl ether and dried in vacuo yielding light greenish to yellow slightly hygroscopic substances in good to excellent yields (>80%).

**(BnEt_3_N)_2_[MnCl_4_],** bis (benzyl-triethylammonium) tetrachloridomanganate(II): Prepared from commercial (BnEt_3_N)Cl. White hygroscopic solid, yield 84%. M.p. (exp.) 106 °C, glass transition (exp.) 13 °C, T_g_/T_m_ = 0.75; elemental analysis for C_26_H_44_MnN_2_Cl_4_ (calcd.): C 54.0 (53.7), H 7.6 (7.6), N 4.9 (4.8); IR (ATR, 32 scans, 25 °C, cm^−1^): 3500, 3444, 3191, 2987, 2944, 1604, 1498, 1480, 1456, 1448, 1480, 1406, 1394, 1371, 1332, 1297, 1210, 1196, 1184, 1155, 1077, 1030, 1009, 941, 903, 808, 777, 752, 708, 699, 613, 604, 547, 537.

**{(Ph_3_P)_2_N}_2_[MnCl_4_]**, bis-(bis (triphenylphosphine) iminium) tetrachloridomanganate(II): Prepared from commercial {(Ph_3_P)_2_N}Cl. White hygroscopic solid, yield 93%. M.p. (exp.) 240 °C, glass transition (exp.) 75 °C, T_g_/T_m_ = 0.68; elemental analysis for C_72_H_60_MnN_2_P_4_Cl_4_ (calcd.): C 66.6 (67.9), H 4.8 (4.8), N 2.3 (2.2); IR (ATR, 32 scans, 25 °C, cm^−1^): 3261, 3054, 2414, 2351, 2141, 1949, 1587, 1435, 1223, 1112, 1104, 1026, 997, 933, 877, 848, 800, 746, 721, 689, 550, 528.

**(EMIm)_2_[MnCl_4_]**, bis (1-methyl-3-methylimidazolium) tetrachloridomanganate(II): Prepared from commercial (EMIm)Cl. Greenish hygroscopic solid, yield 94%. M.p. (exp.) 133 °C (lit. 130 °C [68]), elemental analysis for C_12_H_22_MnN_4_Cl_4_ (calcd.): C 34.6 (34.4), H 5.5 (5.3), N 13.3 (13.4); IR (ATR, 32 scans, 25 °C, cm^−1^): 3140, 3097, 3060, 2960, 2934, 2875, 1738, 1731, 1715, 1651, 1644, 1633, 1614, 1565, 1463, 1446, 1432, 1380, 1338, 1322, 1305, 1280, 1261, 1202, 1164, 1114, 1088, 1067, 1048, 1028, 976, 950, 862, 846, 800, 765, 728, 661, 655, 621.

**(BMIm)_2_[MnCl_4_]**, bis (1-butyl-3-methylimidazolium) tetrachloridomanganate(II): Prepared from (BMIm)Cl [63]. Greenish hygroscopic solid, yield 92%. M.p. (exp.) 67 °C (lit. 62–63 °C [44,47]), elemental analysis for C_16_H_30_MnN_4_Cl_4_ (calcd.): C 40.3 (40.4), H 6.3 (6.4), N 11.6 (11.8); IR (ATR, 32 scans, 25 °C, cm^−1^): 3140, 3097, 3060, 2960, 2934, 2875, 1738, 1731, 1715, 1651, 1644, 1633, 1614, 1565, 1463, 1446, 1432, 1380, 1338, 1322, 1305, 1280, 1261, 1202, 1164, 1114, 1088, 1067, 1048, 1028, 976, 950, 862, 846, 800, 765, 728, 661, 655, 621.

**(DMDIm)[MnCl_4_]**, 3,3′-methylenbis (1-methyl-imidazolium) tetrachloridomanganate(II): Prepared from (DMDIm)Cl_2_ [34]. Greenish hygroscopic solid, yield 80%. M.p. (exp.) 315 °C, elemental analysis for C_9_H_14_MnN_4_Cl_4_ (calcd.): C 28.9 (28.8), H 3.7 (3.8), N 14.8 (14.9); IR (ATR, 32 scans, 25 °C, cm^−1^): 3140, 3097, 3060, 2960, 2934, 2875, 1738, 1731, 1715, 1651, 1644, 1633, 1614, 1565, 1463, 1446, 1432, 1380, 1338, 1322, 1305, 1280, 1261, 1202, 1164, 1114, 1088, 1067, 1048, 1028, 976, 950, 862, 846, 800, 765, 728, 661, 655, 621.

### 2.5. Synthesis of A_2_^+^[Mn(NCO)_4_]^2−^

Samples of the general formula A_2_^+^[Mn(NCO)_4_]^2−^ (A = (BnEt_3_N)^+^, {(Ph_3_P)_2_N}^+^, (BMIm)^+^) were prepared by adaptation of a known literature procedure [42] via salt metathesis reaction of 4.05 eq. freshly precipitated AgOCN (4.05 mmol) and 1.00 eq. A_2_^+^[MnCl_4_]^2−^ (1.00 mmol) in 50 mL boiling nitromethane. The resulting suspensions were refluxed overnight, filtered, afterwards the solvent was removed in vacuo and the residue was washed with portions of dichloromethane. The solvent of the combined filtrates was removed in vacuo yielding light greenish powders or solids in moderate to excellent yields (70–95%).

**(BnEt_3_N)_2_[Mn(NCO)_4_]**, bis (benzyl-triethylammonium) tetraisocyanatomanganate(II): Prepared from (BnEt_3_N)_2_[MnCl_4_] (see above). Light green slightly hygroscopic liquid, yield 73%; elemental analysis for C_30_H_44_MnN_6_O_4_: C 56.6 (58.4), H 7.1 (7.4); N 13.3 (13.6); IR (ATR, 32 scans, 25 °C, cm^−1^): 3503, 2989, 2218, 2172 (CN str. [[69]]), 1712, 1584, 1556, 1498, 1479, 1455, 1395, 1366, 1328 (CO str. [69]), 1300, 1258, 1213, 1184, 1173, 1154, 1100, 1081, 1029, 1006, 942, 902, 857, 830, 791, 753, 704, 656, 622 (NCO bend. [69]), 605, 537.

**{(Ph_3_P)_2_N}_2_[Mn(NCO)_4_]**, bis (bis (triphenylphosphine) iminium) tetraisocyanatomanganate(II): Prepared from {(Ph_3_P)_2_N}_2_[MnCl_4_] (see above). orange solid, yield 95%. M.p. (exp.) 200 °C; elemental analysis for C_68_H_60_MnN_6_O_4_P_4_: C 67.2 (67.8), H 4.7 (5.0), N 7.0 (7.0); IR (ATR, 32 scans, 25 °C, cm^−1^): 3507, 3059, 2219, 2187 (CN str. [69]), 2139, 2046, 1973, 1588, 1563, 1557, 1483, 1437, 1402, 1332 (CO str. [69]), 1297, 1279, 1242, 1177, 1164, 1109, 1074, 1028, 997, 935, 847, 803, 760, 746, 721, 688, 621 (NCO bend. [69]), 552, 528.

**(EMIm)_2_[Mn(NCO)_4_]**, bis (1-methyl-3-methylimidazolium) tetraisocyanatomanganate(II): Prepared from (EMIm)_2_[MnCl_4_] (see above). Light yellow-green liquid, yield 80%. glass transition (exp.) −82 °C, M.p. (calcd.) 13 °C; elemental analysis for C_16_H_22_MnN_8_O_4_ (calcd.): C 43.1 (43.1), H 4.4 (5.0), N 23.9 (25.2); IR (ATR, 32 scans, 25 °C, cm^−1^): 3506, 3148, 3109, 2961, 2935, 2874, 2175 (CN str. [69]), 1713, 1565, 1463, 1427, 1330 (CO str. [69]), 1227, 1164, 1109, 1090, 1023, 948, 842, 751, 698, 651, 619 (NCO bend. [69]).

**(BMIm)_2_[Mn(NCO)_4_]**, bis (1-butyl-3-methylimidazolium) tetraisocyanatomanganate(II): Prepared from (BMIm)_2_[MnCl_4_] (see above). Light green slightly hygroscopic liquid, yield 71%. glass transition (exp.) −80 °C, M.p.b(calcd.) 16 °C; elemental analysis for C_20_H_30_MnN_8_O_4_ (calcd.): C 47.4 (47.9), H 5.5 (6.0), N 20.8 (22.3); IR (ATR, 32 scans, 25 °C, cm^−1^): 3506, 3148, 3109, 2961, 2935, 2874, 2175 (CN str. [69]), 1713, 1565, 1463, 1427, 1330 (CO str. [69]), 1227, 1164, 1109, 1090, 1023, 948, 842, 751, 698, 651, 619 (NCO bend. [69]).

**(DMDIm)[Mn(NCO)_4_]**, 3,3′-methylenbis (1-methyl-imidazolium) tetraisocyanatomanganate(II): Prepared from (DMDIm)[MnCl_4_] (see above). Greenish hygroscopic solid, yield 82%. M.p.(exp.) 132 °C, elemental analysis for C_13_H_14_MnN_4_O_4_ (calcd.): C 38.1 (38.9), H 3.5 (3.5), N 27.4 (27.9); IR (ATR, 32 scans, 25 °C, cm^−1^): 3506, 3148, 3109, 2961, 2935, 2874, 2175 (CN str. [69]), 1713, 1565, 1463, 1427, 1330 (CO str. [69]), 1227, 1164, 1109, 1090, 1023, 948, 842, 751, 698, 651, 619 (NCO bend. [69]).

## 3. Results and Discussion

### 3.1. Syntheses

**Scheme 1:**
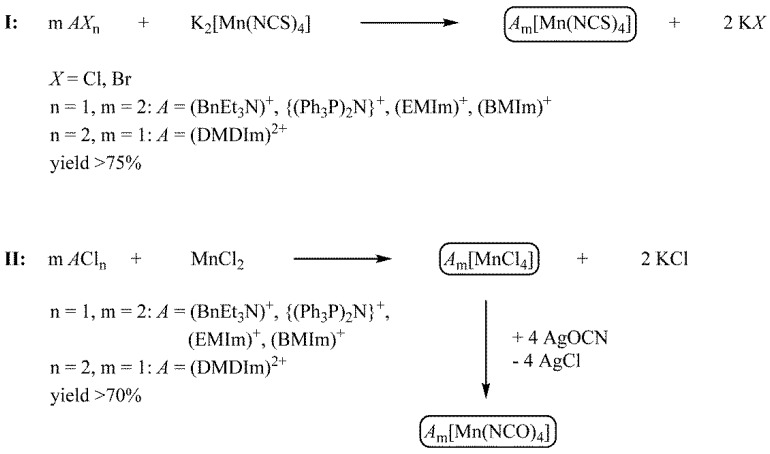
General synthetic approach for the synthesis of [Mn(NCS)_4_]^2−^-based (**I**) and [Mn*X*_4_]^2−^-based salts (*X* = Cl, NCO; **II**).

The schematic synthetic approach to obtain low-melting salts consisting of [Mn*X*_4_]^2−^ complex anions (*X* = Cl, NCS, NCO) is depicted in Scheme 1.

[Mn(NCS)_4_]^2−^-based compounds were synthesized by well-established salt metathesis reactions of ammonium-, iminium- or imidazolium-based halide salts with K_2_[Mn(NCS)_4_] (generated from the reaction of KSCN and MnCl_2_) to generate compounds of the composition A_n_^x+^[Mn(NCS)_4_]^2−^ (n = 2: A = (BnEt_3_N)^+^, {(Ph_3_P)_2_N}^+^, (EMIm)^+^, (BMIm)^+^; n = 1: A = (DMDIm)^2+^) in moderate to high yields (see Scheme 1**I**). The compound with one of the lowest glass transition points, (EMIm)_2_[Mn(NCS)_4_] (T_g_ = −57 °C) was chosen exemplarily for further physicochemical investigations on corresponding densities (*ρ*), dynamic viscosities (*η*), surface tensions (*γ*) and conductivities (*κ*), respectively, and it was synthesized for this purpose at the multi-gram scale (~100–250 mL IL). The reactions were performed in acetonic solutions to ensure complete precipitation of the side product K*X* (*X* = Cl, Br).

[Mn*X*_4_]^2−^-based compounds (*X* = Cl, NCO) were synthesized in a two-step reaction pathway by direct reaction of ammonium-, iminium- or imidazolium-based chloride salts with MnCl_2_ in ethanol solution and further ligand exchange reaction with freshly prepared AgOCN in nitromethane to generate [Mn(NCO)_4_]^2−^-based complexes (see Scheme 1**II**). The respective chlorido- as well as isocyanato complexes show significant tendencies to deliquescence.

### 3.2. Crystal Structures

**Figure 1:**
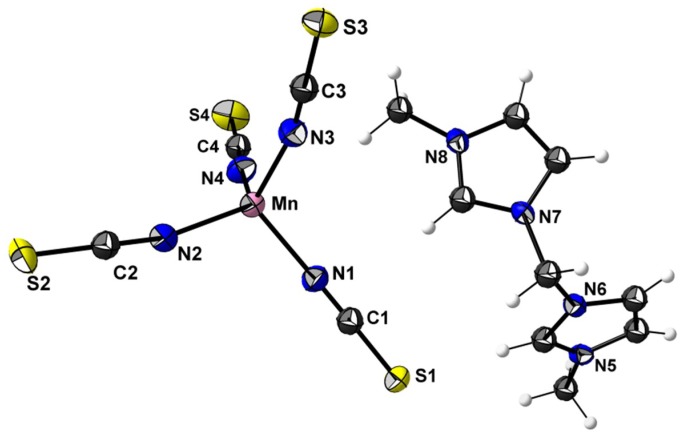
Molecular structure of (DMDIm)[Mn(NCS)_4_] with labeling of selected atoms. Thermal displacement ellipsoids are drawn at the 50% probability level.

**Figure 2:**
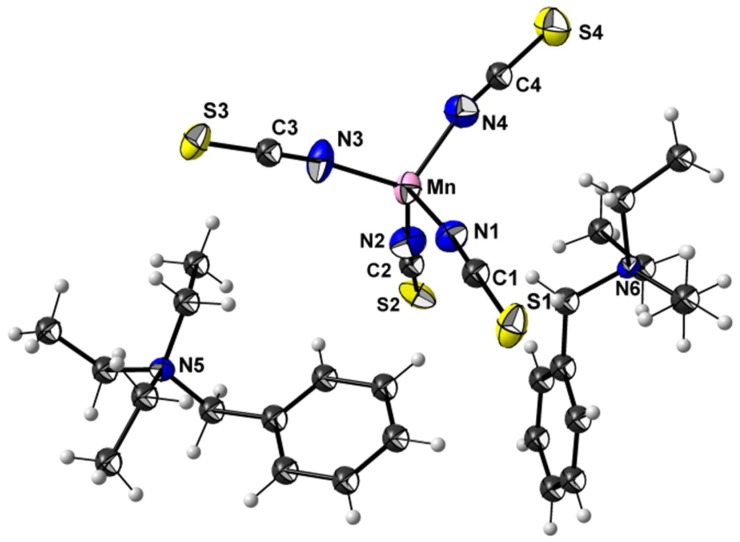
Molecular structure of (BnEt_3_N)_2_[Mn(NCS)_4_] with labeling of selected atoms. Thermal displacement ellipsoids are drawn at the 50% probability level.

**Figure 3:**
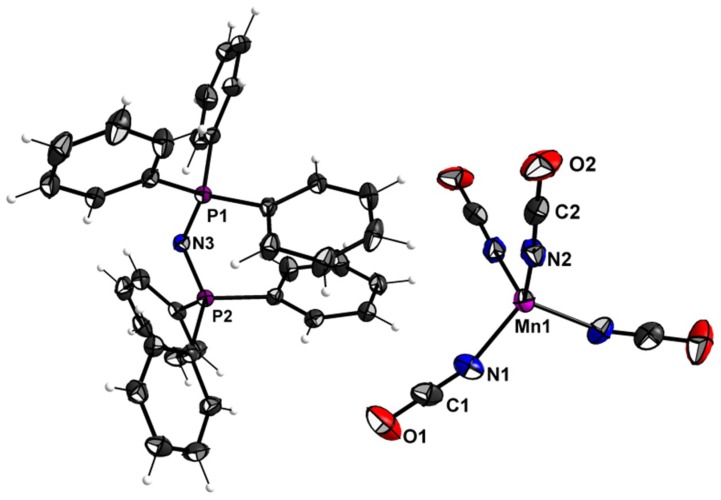
Molecular structure of ((Ph_3_P)_2_N)_2_[Mn(NCO)_4_] with labeling of selected atoms. Thermal displacement ellipsoids are drawn at the 50% probability level.

Single crystals suitable for X-ray structure determinations of (DMDIm)[Mn(NCS)_4_], (BnEt_3_N)_2_[Mn(NCS)_4_], and {(Ph_3_P)_2_N}_2_[Mn(NCO_4_)]·0.6H_2_O were obtained by slow evaporation of the solvents, for example acetonitrile, of solutions of the three compounds at ambient pressure and temperatures of 25 °C. All consist of discrete [Mn(NCX)_4_]^2−^ anions (X = S or O) and the organic N-based cation. Figure 1, Figure 2 and Figure 3 show the molecular structures of the 3 compounds.

All atom distances and angles as well as all the other crystallographic and structure determination parameters are deposited with the CCDC (see Experimental Section). The bond distances and angles are found within the expected ranges. The cations and anions are located on general positions with the exception of the anion in {(Ph_3_P)_2_N}_2_[Mn(NCO)_4_], which is located on the Wyckoff site 2*a* with twofold rotational symmetry. In all cases the Mn atoms have a tetrahedral MnN_4_ coordination environment with some deviations of the N-Mn-N angle from the ideal tetrahedral value. The ranges are 100.82(7)°–112.60(7)° in (DMDIm)[Mn(NCS)_4_], 104.24(8)°–116.83(9)° in (BnEt_3_N)_2_[Mn(NCS)_4_], and 104.2(1)°–113.9(1)° in {(Ph_3_P)_2_N}_2_[Mn(NCO)_4_]. These values average at the ideal tetrahedral angle.

### 3.3. Thermal and Magnetic Properties

Thermal properties of all compounds were measured by using DSC techniques in the temperature range −100 to 600 °C in order to investigate melting points as well as glass transitions. DSC measurements were performed in a three cycle repetition mode. All solid materials were heated above their melting points, then cooled down to −100 °C and again heated up showing in some cases additional glass transitions prior re-crystallization and melting behavior.

The ratio of melting point (T_m_) and glass transition temperature (T_g_) was calculated for those compounds which showed melting and glass transitions in the DSC experiments, for example (DMDIm)[Mn(NCS)_4_]: T_m_ = 179 °C (452.15 K), T_g_ = 26 °C (299,15 K); T_g_/T_m_ = 0.66. These values resemble closely the empirical relationship (T_g_/T_m_ = 2/3 ≈ 0.67 [70,71]) which can be found for a variety of glassy and supercooled systems. The corresponding melting points were calculated on this assumption for all other compounds which showed glass transition behavior only. All observed transitions (melting points and glass transitions) as well as calculated values are listed in detail in the Experimental Section for each compound.

In general, the melting point of candidates from all three subclasses of [Mn*X*_4_]^2−^-based compounds (*X* = Cl, NCS, NCO) can be found below or near the arbitrarily chosen limit for room-temperature ionic liquids (RTILs) of 100 °C, for example (BnEt_3_N)_2_[MnCl_4_]: 106 °C; (BnEt_3_N)_2_[Mn(NCS)_4_]: 101 °C; (BMIm)_2_[Mn(NCO)_4_]: ~16 °C (calcd.). Interestingly, the already relatively low melting point of (BMIm)_2_[MnCl_4_] (67 °C) is even higher by ~17 K than that of (BMIm)_2_[Mn(NCS)_4_] with T_m,calc._ ~50 °C or ~51 K of (BMIm)_2_[Mn(NCO)_4_] with T_m,calc._ ~16 °C. This lowering of the melting point is achieved by exchange from chlorido- to isothiocyanato-, or isocyanato ligands, respectively. The lowest glass transition temperature was detected for (EMIm)_2_[Mn(NCO)_4_] with T_g_ = −82 °C. This behavior is in accordance with comparable [Co(NC*X*)_4_]^2−^-based systems (*X* = S, O) [34].

Magnetic properties at room temperature (T = 25 °C) were measured (EMIm)_2_[Mn(NCS)_4_] and (BMIm)_2_[Mn(NCS)_4_], respectively, by applying NMR techniques [58,59]. Both complexes show magnetic moments in the range of *μ*_eff_ = 5.9–6.0 *μ*_B_ which resembles closely to the spin only value (*μ*_eff_ = 5.92 *μ*_B_) and experimental data of comparable tetrahedral high-spin Mn^II^ complexes, for example (^n^BuPh_3_P)_2_[Mn(NCS)_4_] (*μ*_eff_ = 5.92 *μ*_B_, 296 K [65]), (Ph_2_MeAs)_2_[MnCl_4_] (*μ*_eff_ = 5.88 *μ*_B_, 20 °C [39]), (Et_4_N)_2_[Mn(NCO)_4_] (*μ*_eff_ = 5.98 *μ*_B_, 296 K [42]).

### 3.4. Physicochemical Properties and Influence of Trace Impurities

The impact of water content on physicochemical properties of ILs is known and has been investigated in detail for a long time [72,73,74], but the analyses led to in part opposite results. For example, the basic estimations of contents of water and other impurities in ILs have deficiencies in different applied methods and apparatuses with different and relatively large uncertainties (~10 %). In general, it is known that the influence of water depends strongly of the nature of the ILs and their structures. The situation for viscosities and conductivities is even more complex, but a general trend is known: The higher the water content in aprotic ILs, the lower the viscosities and higher the conductivities. If the water content is below 0.1% an impact of 5%–15% could be detected [72,73]. More recently, the influence of water was also investigated on the speed of sound and antielectrostatic activity in ILs [75,76]. The findings showed that the water content differed significantly from IL to IL at a really low level. The surface tension and densities were not affected significantly and were in agreement with earlier observations [73]. Further insights for such phenomena with regard of the influence of water on properties of ILs were done by theoretical investigations [77].

**Figure 4:**
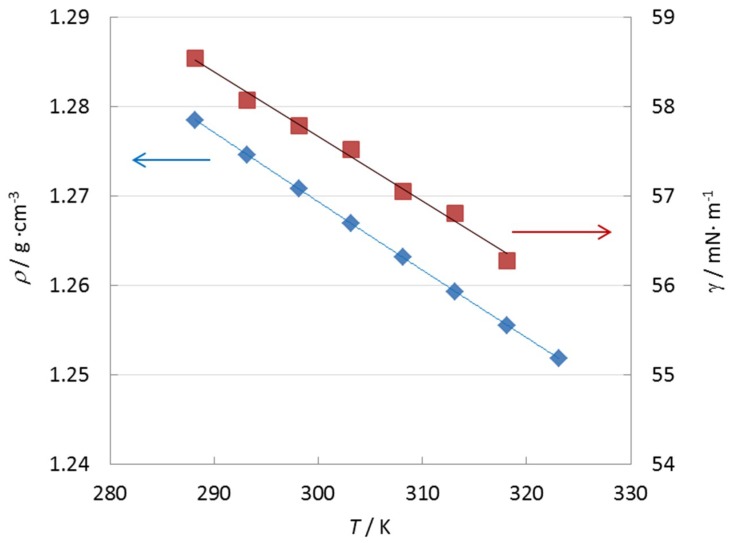
Density (blue squares/line) and surface tension (red squares/line) of (EMIm)_2_[Mn(NCS)_4_] (sample I) at different temperatures T.

**Figure 5:**
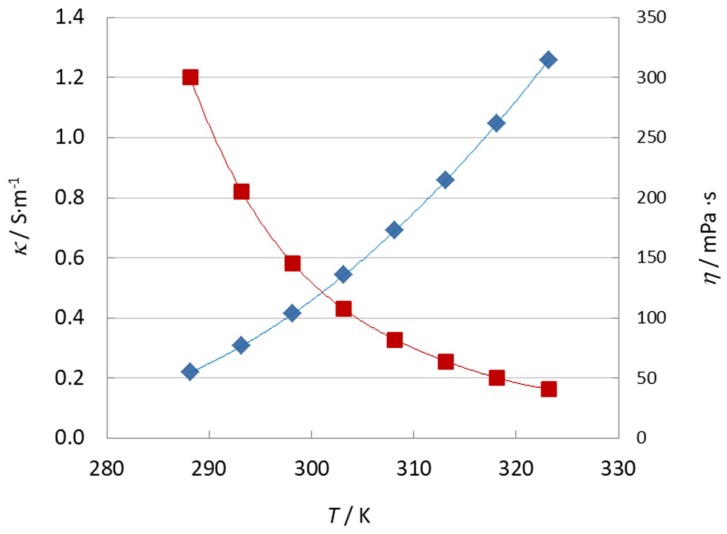
Electrolytic conductivity (blue squares/line) and dynamic viscosity (red squares/line) of (EMIm)_2_[Mn(NCS)_4_] (sample I) at different temperatures T.

The physicochemical properties of three (EMIm)_2_[Mn(NCS)_4_] samples (M = 509.60 g·mol^−1^) with the different residual bromide content (I: 0.006, II: 0.060, III: 0.120 mol·dm^−3^) were investigated for their respective densities (*ρ*), dynamic viscosities (*η*), electrolytic conductivities (κ) and surface tensions (*γ*) in the general temperature range (288.15–323.15) K (step: 5 K). Surface tension measurements were limited to 318.15 K and the water content in all samples was below 200 ppm. From obvious reasons, the results for sample I are discussed in details. Results of all measurements are depicted in Figure 4 and Figure 5, respectively.

The density of all investigated samples is very similar despite of different bromide content and the maximum relative deviation at 298.15 K between samples I and III is −0.3%. The Mn(II)-based ionic liquids have lower density (for sample I: 1.2707 g·cm^−3^ at 298.15 K) than similar ILs with the same cation (EMIm)^+^ and [Co(NCO)_4_]^2−^ or [Co(NCS)_4_]^2−^ anions (1.2981 and 1.2839 g·cm^−3^ at 298.15 K, respectively [25,34].

The maximum deviations for surface tension at 298.15 K between samples I and III is 3.3% and the surface tension of sample I of (EMIm)_2_[Mn(NCS)_4_] (57.8 mN·m^−1^) is similar to the γ value for (EMIm)_2_[Co(NCS)_4_] (55.37 mN·m^−1^) [25].

The agreement between electrolytic conductivity and viscosity for all three investigated samples is very surprising (the maximum deviation is 7.2% and −8.8%, for conductivity and for viscosity, respectively). Furthermore, at 298.15 K the conductivity and dynamic viscosity of (EMIm)_2_[Mn(NCS)_4_] (0.414 S·m^−1^; 145.8 mPa·s) is very similar to comparable values of (EMIm)_2_[Co(NCS)_4_] (0.4 S·m^−1^; 145.4 mPa·s) [25].

In summary, the variation of bromide content on the level investigated in this work does not affect seriously measured parameters. The most interesting fact is that all investigated properties for (EMIm)_2_[Mn(NCS)_4_] mirror the proper values found earlier for (EMIm)_2_[Co(NCS)_4_] [25].

The temperature dependence of the density of the sample I is as follows:

$$\rho /\left(g\cdot {\mathrm{cm}}^{-3}\right)=1.4984-7.63\times 10^{-4}\cdot T/K\;(s=0.7\times 10^{-4}\;g\cdot \mathrm{cm}{}^{-3})$$

where as the temperature dependence of surface tension is:

$$\sigma /\left(\mathrm{mN}\cdot m^{-1}\right)=79.3-72\times 10^{-3}\cdot T/K\;\left(s=0.08\;\mathrm{mN}\cdot m{}^{-1}\right).$$

**Figure 6:**
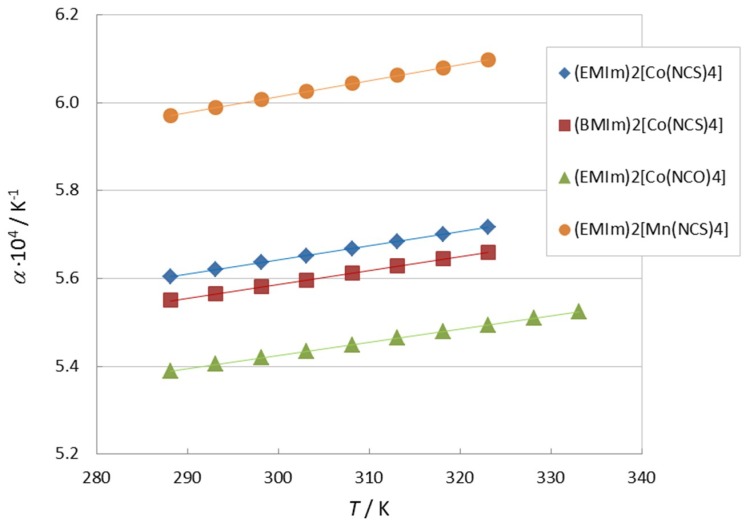
Isobaric coefficient of thermal expansion of (EMIm)_2_[Mn(NCS)_4_] (sample I, orange dots/line) compared to (EMIm)_2_[Co(NCS)_4_] [25] (blue squares/line) and (EMIm)_2_[Co(NCO)_4_] [34] (green triangles/line).

On the basis of the first dependence the isobaric coefficient of thermal expansion, $\alpha_{p}=1/V\cdot {\left(\partial V/\partial T\right)}_{p}=-1/\rho \cdot {\left(\partial \rho /\partial T\right)}_{p}$ was calculated and compared with *α*_p_ for other (EMIm)^+^ salts with [Co(NC*X*)_4_]^2−^ (X = O, S) complex anions (see Figure 6).

*α*_p_ of (EMIm)_2_[Mn(NCS)_4_] is significantly higher than the other regarded (EMIm)^+^-based ionic liquids.

The temperature dependence of surface tension allowed to obtain the surface entropy $S^{a}=-{\left(\partial \gamma /\partial T\right)}_{p},$ which is for (EMIm)_2_[Mn(NCS)_4_] equal 72 × 10^−6^ J·m^−2^·K^−1^. This value is much lower than for other substances taken for comparisons such as (EMIm)_2_[Co(NCO)_4_] with $S^{a}$ = 90.01 × 10^−6^ J·m^−2^·K^−1^ [34] and (EMIm)_2_[Co(NCS)_4_] with $S^{a}$ = 99.4 × 10^−6^ J·m^−2^·K^−1^ [25] and denotes higher surface order for the investigated (EMIm)_2_[Mn(NCS)_4_] IL.

The temperature dependence of viscosity and electric conductivity was described using a Vogel–Fulcher–Tamman (VTF) type of equation [78,79,80]:

$$x=x_{0}\cdot \exp\left(B/\left(T-T_{0}\right)\right)$$

where *x* is *η* (mPa·s) or *κ* (S·m^−1^); *x*_0_-pre-exponential factors *η*_0_ and *κ*_0_ (in units as regarded transport quantities); *B* is a material constant and *T*_0_ is temperature of an ideal glass transition. Fitting parameters *x*_0_, *B* and *T*_0_ are collected in Table 1.

The value of *T*_0_ obtained for (EMIm)_2_[Mn(NCS)_4_] (sample I) from conductivity (see Table 1) agrees very well with the value from viscosity. Generally, the temperature of an ideal glass transition for all (EMIm)^+^-based ionic liquids calculated based on temperature dependence of conductivity are very close (208(5) K and 206.5(1.4) K for (EMIm)_2_[Co(NCS)_4_] and (EMIm)_2_[Co(NCO)_4_], respectively) [25,34]. However, *T*_0_ calculated from viscosity reveals some kind of variation: It is the lowest for (EMIm)_2_[Co(NCS)_4_] (172(12) K [25]) and higher for (EMIm)_2_[Co(NCO)_4_] (190.4(0.2) K [34]). Thus, the highest *T*_0_ from viscosity has (EMIm)_2_[Mn(NCS)_4_] (sample I) investigated in this work.

## Figures and Tables

**Table 1:** Fitting parameters of Vogel–Fulcher–Tamman (VTF) equations applied to the viscosity and the electrolytic conductivity of (EMIm)_2_[Mn(NCS)_4_] (sample I).

***η*_0_/(mPa·s)**	***B*/K**	***T*_0_/K**	***r***
0.348 ± 0.034	570 ± 20	204 ± 2	0.999996
***κ*_0_/(S·m^−1^)**	***B*/K**	***T*_0_/K**	***r***
72.4 ± 4.5	−471 ± 13	206.9 ± 1.4	0.999996
